# Buerger’s Disease as a Cause of Post-Operative Skin Necrosis: A Case Report

**DOI:** 10.3390/jpm13010108

**Published:** 2023-01-03

**Authors:** Jong Hyup Kim, Hoon Kim, In Chang Koh, Soo Yeon Lim

**Affiliations:** Department of Plastic and Reconstructive Surgery, Myunggok Medical Research Institute, Konyang University Hospital, College of Medicine, Konyang University, Daejeon 35365, Republic of Korea

**Keywords:** necrosis, postoperative complications, smoking cessation, thromboangiitis obliterans, wound healing

## Abstract

Postoperative skin necrosis occurs due to various causes, including infections, lack of oxygenation, underlying diseases, and lack of nutrition. Therefore, a thorough evaluation of the cause of skin necrosis should be conducted. In the present case, the patient underwent disarticulation of the interphalangeal joint of the right first toe by an orthopedic surgeon and developed postoperative skin necrosis. Through physical examination, imaging studies, and detailed medical history evaluation, the patient was diagnosed with thromboangiitis obliterans, also known as Buerger’s disease. After 4 months of medication for Buerger’s disease and smoking cessation, the wound healed without complications or recurrence. Therefore, in cases of untreatable chronic wounds in young and heavy smoking patients, Buerger’s disease should be suspected as a potential cause.

## 1. Introduction

Postoperative skin necrosis occurs due to various causes, such as infections, pressure ulcers, and vascular disorders. Treatment for the cause of skin necrosis is essential for a successful recovery; thus, a proper evaluation to determine the cause is important.

Buerger’s disease is a non-atherosclerotic, segmental inflammatory disease of small arteries and veins, with secondary thrombosis and partial or total vascular obstruction [1,2]. This usually results in symptoms such as distal extremity ischemia, ischemic ulcers, and gangrene, and often causes skin necrosis [1,3]. We report a case where delayed wound healing was appropriately treated by diagnosing the patient with Buerger’s disease, which was initially overlooked owing to the patient’s young age and having no underlying diseases.

## 2. Case Presentation

**Figure 1:**
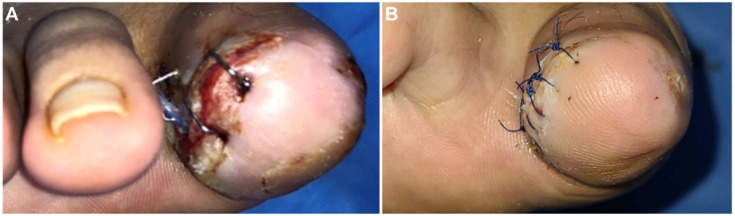
(**A**) Postoperative skin necrosis after interphalangeal joint disarticulation of right first toe (7 days after disarticulation). On the lateral side of the right first toe, skin necrosis with dehiscence occurred, and wound healing was delayed; (**B**) The skin was well epithelialized without any complication after debridement and wound repair with proper medication, smoking cessation, and persistent aseptic dressing with local oxygenation therapy (8 days after debridement and wound repair).

A 36-year-old man was admitted to our hospital with osteomyelitis after surgery for an ingrown nail of the right first toe at another hospital. At the Department of Orthopedics, the patient underwent disarticulation of the interphalangeal joint of the right first toe. After disarticulation, the wound was dressed using ordinary methods and closely monitored. However, wound healing was delayed for over a month, and wound disruption and skin necrosis occurred (Figure 1A). Subsequently, a consultation for plastic surgery was requested, and the patient was transferred. Debridement and wound repair of the right first toe was performed with antibiotic treatment. Despite persistent negative findings in the wound culture and no signs of wound infection, the wound healed very slowly.

**Figure 2:**
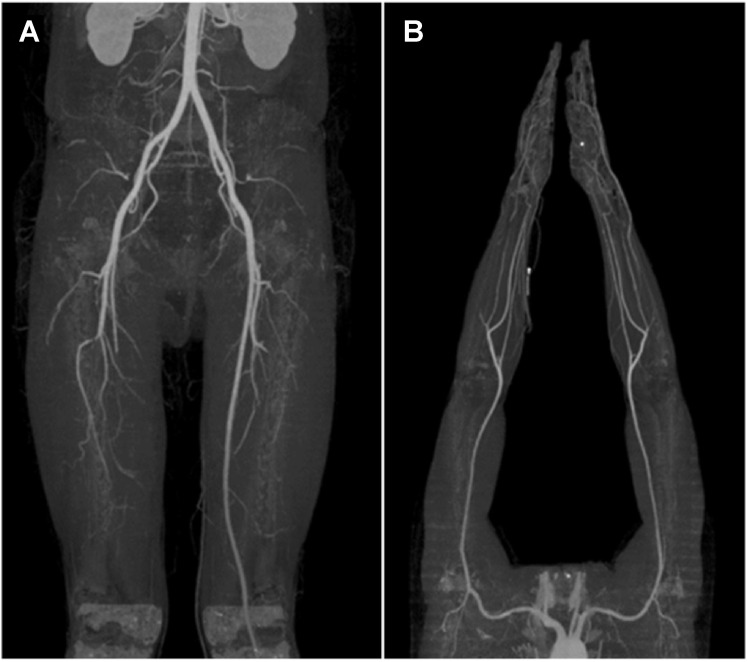
(**A**) Lower extremity computed tomography angiography showing abrupt occlusion of the right popliteal artery; (**B**) Upper extremity computed tomography angiography showing total occlusion at both distal ulnar arteries with corkscrew collaterals.

Upon physical examination, the dorsalis pedis artery was hardly palpable, and poor blood flow was confirmed through handheld Doppler examination. Based on these findings, and considering that the patient was a young man with a smoking history of >20 packs per year, thromboangiitis obliterans, known as Buerger’s disease, was suspected. Upper- and lower-extremity computed tomography angiography was performed for confirmation; the related findings showed abrupt occlusion of the right popliteal artery (Figure 2A) and both ulnar arteries with corkscrew collaterals (Figure 2B). The patient was diagnosed with Buerger’s disease, and percutaneous transluminal angioplasty (PTA) was performed after consultation with a cardiologist.

Laboratory findings showed nonspecific results, with erythrocyte sedimentation rate (ESR) = 5 mm/h, C-reactive protein (CRP) = 0.1 mg/dL (normal range 0.1–0.5 mg/dL), and HbA1C 5.4%. Other typical laboratory test results were also within a normal range. Chest X-ray and standard Electrocardiogram findings were normal. Immunologic tests showed negative results with anti-phospholipid antibody <1.0 U/mL and anti-cardiolipin antibody <3.0 immunoglobulin G [IgG] phospholipid U/mL.

**Figure 3:**
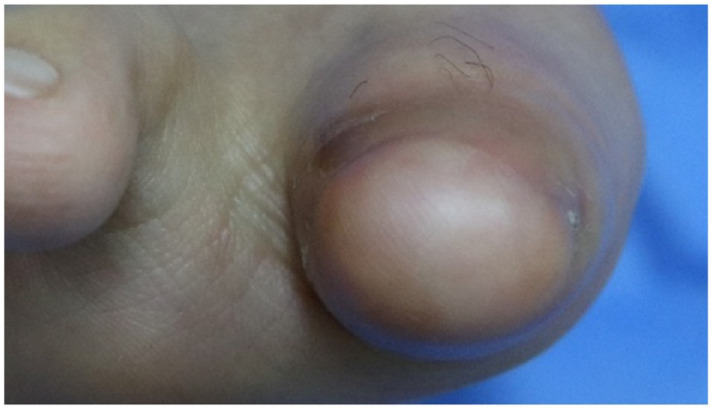
Four-month follow-up images showing satisfactory recovery without serious complications.

After consultation with a vascular surgeon, the patient was advised to take cilostazol along with medications for Buerger’s disease, including alprostadil (Eglandin), beraprost, and nafronyl oxalate. Medication, smoking cessation, necrotic tissue debridement, wound repair, and persistent aseptic dressing with local oxygenation therapy (Figure 1B) were performed; the necrotic tissue biopsy results were of simple ‘necrosis’. The skin epithelialized well without complications, and the stitch was removed 14 days after wound repair. In subsequent outpatient observations, the wound fully recovered and was maintained (Figure 3). The patient provided informed consent for the publication of this case and related imaging findings. Written informed consent was obtained for publication of this case report and accompanying images.

## 3. Discussion

Many factors affect wound healing, such as oxygenation, infections, diabetes, medications, smoking, and lack of nutrition. If these factors are not properly regulated, post-operative complications such as wound rupture, anastomotic leakage, skin necrosis, epidermolysis, and decreased wound tensile strength may occur [4]. Therefore, an evaluation of the fundamental causes of skin necrosis should be performed for postoperative treatment of complications.

In the present case, despite providing an optimal environment for wound healing, such as empirical antibiotic treatment, periodic dressing treatment, and hyperbaric treatment after disarticulation of the interphalangeal joint of the right first toe, the wound hardly healed, and skin necrosis developed [4]. The factors affecting wound healing that should be considered are a moist environment, signs of infection, pressure on the wound interfering with blood supply, presence of chronic underlying diseases, and immunosuppressive treatment [4,5]. In this case, the surgeon who initially administered the treatment overlooked the vascular problems because the patient was young and had no underlying conditions. Moreover, the patient’s clinical history and the findings on initial physical examination showed no signs indicative of Buerger’s disease. Additionally, there were no signs of critical limb ischemia, such as intermittent claudication or pain. Therefore, Buerger’s disease was initially overlooked. However, considering that the patient was a heavy smoker and wound healing was delayed, despite no evident underlying disease, we suspected Buerger’s disease. Physical examinations were performed with pulse palpation of blood vessels and a handheld Doppler; blood flow in the lower extremities, such as the dorsalis pedis and posterior tibial artery, were assessed to confirm vascular problems [3,6].

Buerger’s disease can be diagnosed when young smokers have signs of peripheral ischemia, recurrent superficial thrombophlebitis involving the upper extremities, and no signs of diabetes or atherosclerosis [2,7]. Thus, Buerger’s disease may be suspected if a patient is a heavy smoker and no other factors that may interfere with wound healing are present, such as thrombophilia, autoimmune disease, diabetes, and a proximal source of emboli [2,3]. In Buerger’s disease, distal vessel disease without calcification, segmental occlusive lesion, or development of corkscrew collaterals are commonly found during a computed tomography angiography [2,3,8,9]. In our case, the patient was diagnosed with Buerger’s disease based on medical history, physical examination, and imaging tests.

According to previous reports, smoking cessation is the most effective treatment for Buerger’s disease [2,3,8,9]. Complete smoking cessation can prevent wound necrosis progression and reduce the probability of amputation [8,9]. Therefore, physicians should educate patients to stop using tobacco products and avoid exposure to secondhand smoke [3,8,10]. Other recommended treatments include surgical revascularization, such as bypass surgery, in patients with severe ischemia, although its effectiveness for ischemic ulcers of Buerger’s disease is controversial [3,8,9]. Similarly, endovascular therapy, such as PTA, may also be helpful [8,9]. In terms of medications, prostaglandin analogues such as iloprost and aspirin can be considered and, in patients with ischemic pain, vasodilators such as calcium channel blockers, sildenafil, alpha-blockers, and cilostazol may be helpful [3,8,10]. In our case, the patient was prescribed alprostadil, beraprost, and nafronyl oxalate after consultation with a vascular surgeon. Additionally, after consultation with a cardiologist, PTA was performed to dilate the narrowed anterior and posterior tibial arteries, and cilostazol was prescribed. Importantly, necrotic tissue debridement and wound repair were performed while dressing treatment was continued (Figure 1B). As a result, the wound healed quickly and showed good progress without complications or recurrence.

This case showed the healing process of a wound by distinguishing vascular disease among various factors that affect wound healing when postoperative skin necrosis occurs. Among various vascular diseases, Buerger’s disease was suspected and diagnosed through appropriate evaluation, and the wound healed completely. As such, if wounds are not appropriately healed even though there is no underlying disease that could hinder wound healing, young patients with a history of heavy smoking should be suspected of Buerger’s disease and evaluated accordingly and appropriately.

## Data Availability

Not applicable.

## References

[1] Kim D.Y., Mun G.H. (2012). A treatment of open wound due to Buerger’s disease using Healoderm: Case report. J. Korea Wound Manag. Soc..

[2] Elfarra M., Rădulescu D., Peride I., Niculae A., Ciocâlteu A., Checheriţă I.A., Lascăr I., Sinescu D.R. (2015). Thromboangiitis obliterans-Case report. Chirurgia.

[3] Piazza G., Creager M.A. (2010). Thromboangiitis obliterans. Circulation.

[4] Guo S., Dipietro L.A. (2010). Factors affecting wound healing. J. Dent. Res..

[5] Hess C.T. (2011). Checklist for factors affecting wound healing. Adv. Skin Wound Care.

[6] Khan N.A., Rahim S.A., Anand S.S., Simel D.L., Panju A. (2006). Does the clinical examination predict lower extremity peripheral arterial disease?. JAMA.

[7] Akar A.R., Durdu S., Hoffman G.S., Weyand C.M., Langford C.A., Goronzy J.J. (2012). Buerger’s disease (thromboangiitis obliterans). Inflammatory Diseases of Blood Vessels.

[8] Del Conde I., Peña C. (2014). Buerger disease (thromboangiitis obliterans). Tech. Vasc. Interv. Radiol..

[9] Kim S.H., Choi H.J., Park E.S. (2016). The wound healing effects of percutaneous transluminal angioplasty for an entire dorsal foot ulcer with Buerger’s disease: Prevention of major amputation. J. Korea Wound Manag. Soc..

[10] Arkkila P.E. (2006). Thromboangiitis obliterans (Buerger’s disease). Orphanet J. Rare Dis..

